# Dr. Milton Almond Curtiss

**Published:** 1916-05

**Authors:** 


**Dr. Milton Almond Curtiss,** Syracuse 1878, died at his home in Kirkville, Feb. 27, of pneumonia following grippe, aged 61. He was the father of Dr. Carlton C. Curtiss of Rochester.

